# Overexpression of ABCC1 Confers Drug Resistance to Betulin

**DOI:** 10.3389/fonc.2021.640656

**Published:** 2021-02-25

**Authors:** Xuan-Yu Chen, Yuqi Yang, Jing-Quan Wang, Zhuo-Xun Wu, Jing Li, Zhe-Sheng Chen

**Affiliations:** ^1^ College of Integrated Chinese and Western Medicine, Hebei Medical University, Shijiazhuang, China; ^2^ Department of Pharmaceutical Sciences, College of Pharmacy and Health Sciences, St. John’s University, New York, NY, United States

**Keywords:** betulin, ATP-binding cassette sub-family C member 1 (ABCC1), multidrug resistance-associated protein 1 (MRP1), multidrug resistance (MDR), natural product

## Abstract

Betulin is a lupane-type pentacyclic triterpene, which is isolated from birch bark. It has a broad spectrum of biological and pharmacological properties, such as anti-inflammatory, anti-tumor, anti-viral, and anti-bacterial activity. Herein, we explored the factors that may result in betulin resistance, especially with respect to its interaction with ATP-binding cassette subfamily C member 1 (ABCC1). ABCC1 is an important member of the ATP-binding cassette (ABC) transporter family, which is central to mediating multidrug resistance (MDR) in naturally derived anticancer agents. An MTT-based cell viability assay showed that ABCC1 overexpression has the ability to desensitize both cancer cell line and gene-transfected cell line to betulin and that this betulin-induced resistance can be antagonized by a known ABCC1 inhibitor MK571 at 25 μM. Additionally, betulin upregulates the ABCC1 protein expression level in both concentration-dependent and time-dependent manners, also blocks the transport function mediated by ABCC1. Subsequently, a high affinity score of betulin was achieved in a computational docking analysis, demonstrating a strong interaction of betulin with ABCC1.

## Introduction

To date, many drugs have originated from natural products, including vincristine, vinblastine, doxorubicin, and paclitaxel all have the potential to inhibit tumor progression (1). However, the effectiveness of these drugs can be restricted by multidrug resistance (MDR)-associated ATP-binding cassette (ABC) transporters (2). Specifically, vincristine, vinblastine, doxorubicin, and paclitaxel can be transported by ABC sub-family B member 1 (ABCB1, multidrug resistance protein 1/MDR1, P-glycoprotein/P-gp) (3); whereas, vincristine, vinblastine, and doxorubicin are substrate drugs of ABC sub-family C member 1 (ABCC1, multidrug resistance protein 1/MRP1) (4).

The 190 kDa MRP1, located in human chromosome locus *p13.11*, was firstly isolated from doxorubicin-resistance small cell lung cancer line H69AR, and was found to be associated with MDR in 1992 (5, 6). The protein structure of MRP1 has three membrane-spanning domains (MSDs), and two nucleotide-binding domains (NBDs) (7). Functionally, the NBDs serve as the energy source to produce hydrolyzed ATP, while the MSDs provide support for drug binding, putative drug transport channel, dimerization, and trafficking (4). MRP1 has a wide distribution, for example throughout the adrenal gland, bladder, choroid plexus, helper T cells, and muscle cells (8).

**Figure 1:**
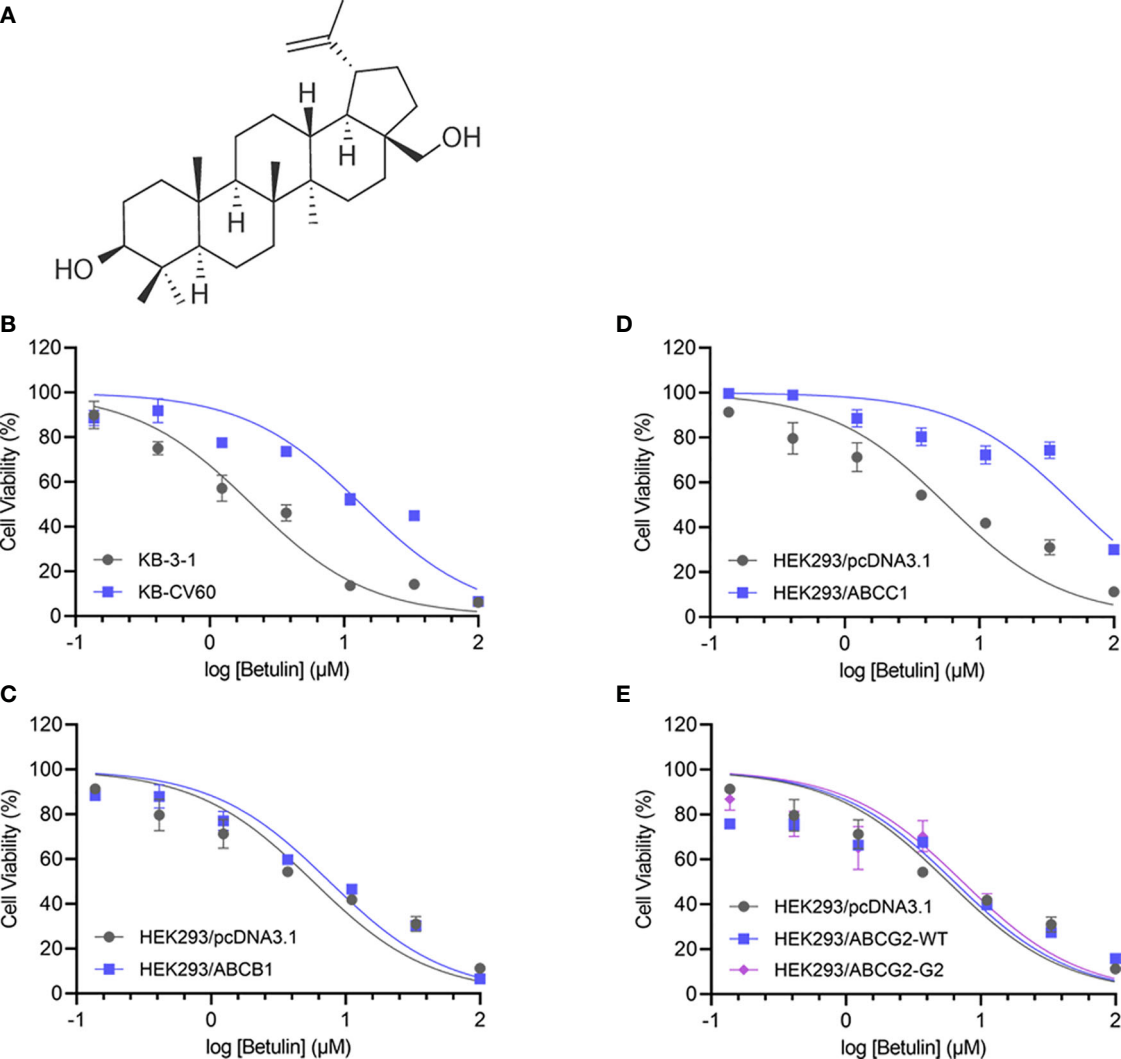
Chemical structure and cell viability-concentration curves. **(A)** The chemical structure of betulin. The cell viability-concentration curves for **(B)** KB-3-1 and KB-CV60 cells, **(C)** HEK293/pcDNA3.1 and ABCC1-tansfected HEK293 cells, **(D)** HEK293/pcDNA3.1 and ABCB1-tansfected HEK293 cells, **(E)** HEK293/pcDNA3.1 and HEK293 cells transfected with wild-type or R482G-mutant ABCG2 after treatment with serial concentrations of betulin. Each point is shown as mean ± SD, from the experiment that was repeated at least three times and performed independently.

Betulin, a lupane-type pentacyclic triterpene, is isolated from bark of birches. Due to its poor solubility in aqueous media, several more soluble derivatives and betulin nanoparticles were developed (9, 10). The chemical structure of betulin is presented in **Figure 1A**. Betulin and its derivatives have a broad spectrum of anti-cancer profile, such as against lung, breast, prostate, colon, colorectal, cervical, and pancreatic cancer, as well as melanoma and leukemia (11). The interaction between betulinic acid and ABCB1 or ABCG2 is conclusive (12, 13); however, the effect of ABCC1 on betulin efficacy needs to be elucidated. Interestingly, it has been documented that ABCC1 overexpression is correlated with lung, breast, prostate, and ovarian cancer, gastrointestinal carcinoma, melanoma, and leukemia (14, 15). Based on the overlapping cancer spectrum, we postulated that the overexpression of ABCC1 may attenuate the anticancer efficacy of betulin.

Herein, we focused on the interaction of betulin with ABCC1, and found that ABCC1 overexpression confers drug resistance to betulin. This finding may provide a valuable foundation for future preclinical and clinical investigations of betulin.

## Materials and Methods

### Chemicals and Reagents

Betulin was kindly provided as a free sample from MedChemExpress (Purity > 98.0%, Monmouth Junction, NJ). Dulbecco’s modified Eagle medium, trypsin-EDTA, penicillin/streptomycin, and fetal bovine serum were purchased from Corning (Corning, NY). Phosphate buffer saline (PBS) was obtained from VWR chemicals (Solon, OH). Vincristine, dimethyl sulfoxide (DMSO), and methylthiazolyldiphenyl-tetrazolium bromide (MTT) were obtained from Millipore-Sigma (Burlington, MA). MK571 and G418 were purchased from Enzo Life Sciences (Farmingdale, NY). Anti-MRP1/ABCC1 (D5C1X) antibody (product #72202), anti-GAPDH (D16H11) antibody (product #5174), and HRP-conjugated anti-rabbit IgG secondary antibody (product #7074) were obtained from Cell Signaling Technology (Danvers, MA). [^3^H]-Vincristine (0.7 Ci/mmol) was purchased from Moravek Biochemicals (Brea, CA). Liquid scintillation cocktail, and all other chemicals and reagents were purchased from ThermoFisher Scientific (Waltham, MA).

### Cell Lines and Cell Culture

Human epidermoid carcinoma cell line KB-3-1 was used as the drug-sensitive cell line, and its ABCC1-overexpressing cell line KB-CV60 was maintained in the medium with 1 µg/ml of cepharanthine and 60 ng/ml of vincristine (16). HEK293/pcDNA3.1, HEK293/ABCC1, HEK293/ABCB1, HEK293/ABCG2-WT, and HEK293/ABCG2-G2 were transfected with either an empty vector pcDNA3.1 or a pcDNA3.1 vector containing a full length ABCC1, ABCB1, or ABCG2 encoding arginine (R) or glycine (G) at position 482 (17). All transfected cells were selected in the medium with 2 mg/ml G418. All cell lines were cultured in medium supplemented with 10% serum and 1% antibiotics at 37°C under 5% CO_2_. All drug resistant cell lines were cultured in drug-free medium for more than 2 weeks prior to further use.

### MTT-Based Cell Viability Assay

An established protocol of MTT assay was used to determine the cell viability of betulin with or without an inhibitor (18). DMSO was used as a solvent to prepare a stock solution (10 mM) of all compounds. As the highest concentration in the cell viability assay was 100 μM, the final concentration of DMSO was 1% in the treatment medium. Also, all control groups in the experiment were treated with solvent only. Briefly, 5,000 to 7,000 cells/well were evenly seeded in a 96-well plate, and incubated at 37°C overnight prior to further experiment. On the next day, a serial dilution of substrate drugs (0–100 μM) with or without inhibitors at the indicated concentration was added to the designated well. The cells were further incubated for 72 h. On the last day of treatment period, MTT solution was added to each well, and incubated for 4 h protected from light. After incubation, the supernatant was removed, and 100 μl/well DMSO was added to dissolve the formazan crystals. Subsequently, absorbance at 570 nm was measured by an UV/Vis microplate spectrophotometer (Fisher Science, Fair Lawn, NJ). The log scale curves in GraphPad (log inhibitor vs. responses) were used to fit cell viability curves and to calculate the IC_50_ values. The values of resistance fold (RF) were calculated as previously stated (19).

### [^3^H]-Vincristine Accumulation Assay

The transport function of MDR-associated ABC transporter was determined by tritium-labeled substrate accumulation assay (20). Briefly, 5×10^5^ cells/well were seeded evenly in a 24-well plate, and incubated for 24 h. On the second day, cells were treated with betulin or MK571 at the indicated concentrations for 2 h. Then, [^3^H]-vincristine was added to the designated wells at a final concentration of 36 nM. After 2 h incubation, cells were washed with PBS twice, and harvested and transferred into scintillation fluid. Subsequently, the intracellular radioactivity was measured by a liquid scintillation analyzer (Packard Instrument, Downers Grove, IL).

### Western Blot Analysis

As previously described, the protein expression level of ABCC1 was examined by a Western blot analysis (19). In short, cells were treated with or without betulin, and then the lysate was collected. This was followed by determining the protein concentration in the lysates, and equal amount of protein sample (12 μg) was separated by SDS-PAGE then transferred onto PVDF membrane. The membranes were blocked for 2 h with 5% non-fat milk at room temperature followed by incubation with primary antibody (anti-MRP1/ABCC1 and anti-GAPDH at 1:1000) overnight at 4°C. On the second day, after washing with TBST three times, the membranes were incubated with HPR-conjugated secondary antibody (at 1:1000) for 1 h at room temperature. Subsequently, the protein was visualized using an ECL substrate by a digital Western blot scanner (LI-COR, NE), and quantified and analyzed by Fiji software (NIH, Bethesda, MD).

### Molecular Docking Analysis of Betulin With Human ABCC1 Model

The betulin 3D structure was constructed for docking simulation with an ABCC1 model as previously described (21). ABCC1 protein model 5UJA (LTC_4_ bound) was obtained from RCSB Protein Data Bank. The model is inward-facing ABCC1 with a resolution of 3.34 (22). Docking calculations were performed in AutoDock Vina (version 1.1.2) (23). Hydrogen atoms and partial charges were added using AutoDock Tools (ADT, version 1.5.4). Docking grid center coordinates were determined from the bound ligand LTC_4_ provided in 5UJA PDB files. Receptor/ligand preparation and docking simulation were performed using default settings. The top-scoring pose (sorted by affinity score: kcal/mol) was selected for further analysis and visualization.

### Statistics

All data were presented as mean ± SD, and evaluated using a one-way or two-way ANOVA as appropriate by GraphPad software (La Jolla, CA). Statistical significance was considered when *p* < 0.05.

## Results

### The Efficacy of Betulin Is Attenuated in ABCC1-Overexpressing Cells and That This Betulin-Induced Resistance Can Be Sensitized by ABCC1 Inhibitor

An MTT assay was performed to examine the cell viability of substrate drugs with or without an inhibitor. Herein, the RF values were used to assess the degree of attenuated effectiveness resulting from ABCC1 overexpression. As shown in **Figures 1B, C**, the efficacy of betulin was restricted in cells expressing ABCC1 as evidenced by higher IC_50_ values in MDR cells mediated by ABCC1 compared to their corresponding drug-sensitive cell line counterparts. The RF value of betulin was significantly increased to 6.53-fold in KB-CV60 cells, and 8.93-fold in ABCC1-transfected HEK293 cells (**Table 1**). Importantly, the betulin-induced drug resistance can be antagonized by a known ABCC1 inhibitor MK571 at 25 μM. By contrast, vincristine serves as a reference substrate to compare the drug resistance conferred by ABCC1. Based on **Table 1**, vincristine had 19.33- and 23.79-fold resistance in drug-selected cancer cells and gene-transfected cells expressing ABCC1, and similarly, this resistance can be reversed by MK571 at 25 μM. Thus, betulin is less sensitive in ABCC1-overexpressing cells, and an established ABCC1 inhibitor could overcome the resistance effect induced by betulin.

**Table 1:** MK571 at 25 μM sensitized ABCC1-overexpressing cells to betulin.

Treatment	IC_50_ ± SD^a^ (RF^b^) (μM)			
	KB-3-1	KB-CV60	HEK293/pcDNA3.1	HEK293/MRP1
Betulin	2.06 ± 0.29 (1.00)	13.47 ± 0.89 (6.53)^*^	5.73 ± 0.71 (1.00)	51.06 ± 4.25 (8.92)^*^
+ MK571 25 μM	3.34 ± 0.42 (1.62)	6.94 ± 0.61 (3.36)	5.36 ± 0.42 (0.94)	22.33 ± 1.57 (3.90)
Vincristine	0.02 ± 0.01 (1.00)	0.29 ± 0.03 (19.33)^*^	0.39 ± 0.02 (1.00)	9.28 ± 1.04 (23.79)^*^
+ MK571 25 μM	0.02 ± 0.01 (1.47)	0.10 ± 0.01 (6.53)	0.47 ± 0.03 (1.21)	2.44 ± 0.41 (6.26)

a The half-maximal inhibitory concentration (IC_50_) values are showed as mean ± SD from at least three independent experiments performed in triplicate.

b Resistance fold (RF) was calculated by dividing the IC_50_ values of drug-sensitive cells with MK571 or cells expressed ABCC1 with or without MK571 by the IC_50_ values of their corresponding parental cells without MK571.

^*^p < 0.05 versus control group.

### ABCB1 or ABCG2 Cannot Confer Resistance to Betulin in Cells Overexpressed ABCB1 or ABCG2

As overexpression of ABCB1 and ABCG2 are also central to MDR, cell lines expressing either ABCB1 or ABCG2 were investigated. Based on **Figures 1D, E**, the IC_50_ values of betulin were 5.72, 7.56, 6.37, and 7.34 μM for HEK293/pcDNA3.1, HEK293/ABCB1, HEK293/ABCG2-WT, and HEK293/ABCG2-G2 cells, respectively. The RF values were 1.32, 1.11, and 1.28 in ABCB1-overexpressing cells, and wide-type or R482G-mutant ABCG2-overexpressing cell lines, respectively (**Figures 1D, E**). Given no significant difference in RF values between drug-sensitive and MDR cells mediated by ABCB1 or ABCG2, it is likely that ABCB1 and ABCG2 overexpression did not affect the effectiveness of betulin.

### Betulin Blocks the Transport Function Mediated by ABCC1

**Figure 2:**
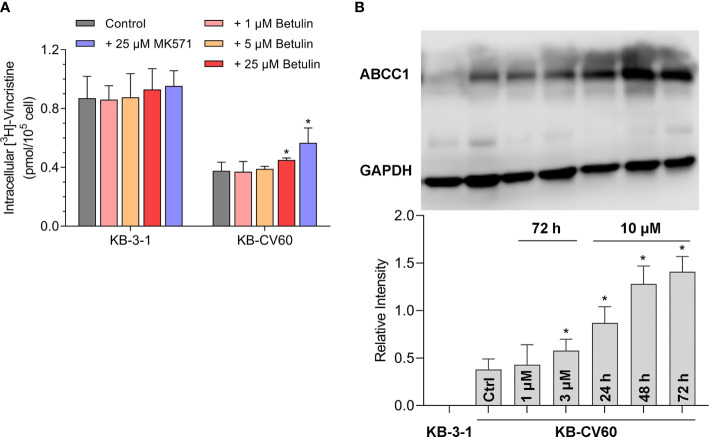
Effects of betulin on the transport function mediated by ABCC1 and the ABCC1 protein expression level. **(A)** [^3^H]-vincristine intracellular accumulation in KB-3-1 and KB-CV60 cells after betulin or MK571 treatment at indicated concentrations. **(B)** The ABCC1 protein expression level after incubated with 1 μM or 3 μM betulin for 72 h, or with 10 μM betulin up to 72 h. All data are presented as mean ± SD. *indicates *p* < 0.05 compared with its corresponding control group.

A tritium-labeled drug accumulation analysis was conducted to assess the interaction between betulin and MDR-associated ABC transporter. Our results in **Figure 2A** showed that betulin at 25 μM enhanced the intracellular vincristine accumulation from 44% to 52% compared with KB-CV60 cells without an inhibitor. Herein, 25 μM MK571 acted as a reference ABCC1 inhibitor to increase substrate drug accumulation in KB-CV60 cells (from 44% to 65%). Therefore, betulin at a high concentration has the ability to impede the ABCC1 transport function resulting in increased level of intracellular accumulation of substrate drug.

### Betulin Induces ABCC1 Protein Expression Level

It is documented that upregulation of ABC transporter expression can induce MDR. A Western blot analysis was used to determine the ABCC1 protein expression level after betulin treatment. As shown in **Figure 2B**, 3 μM betulin had the ability to significantly induce ABCC1 expression after a 72 h incubation period. Also, the expression level of ABCC1 time-dependently increased following treatment with betulin at 10 μM up to 72 h. Betulin time- and concentration-dependently upregulated ABCC1 protein expression.

### Docking Simulation of Betulin in the Drug-Binding Pocket of ABCC1

**Figure 3:**
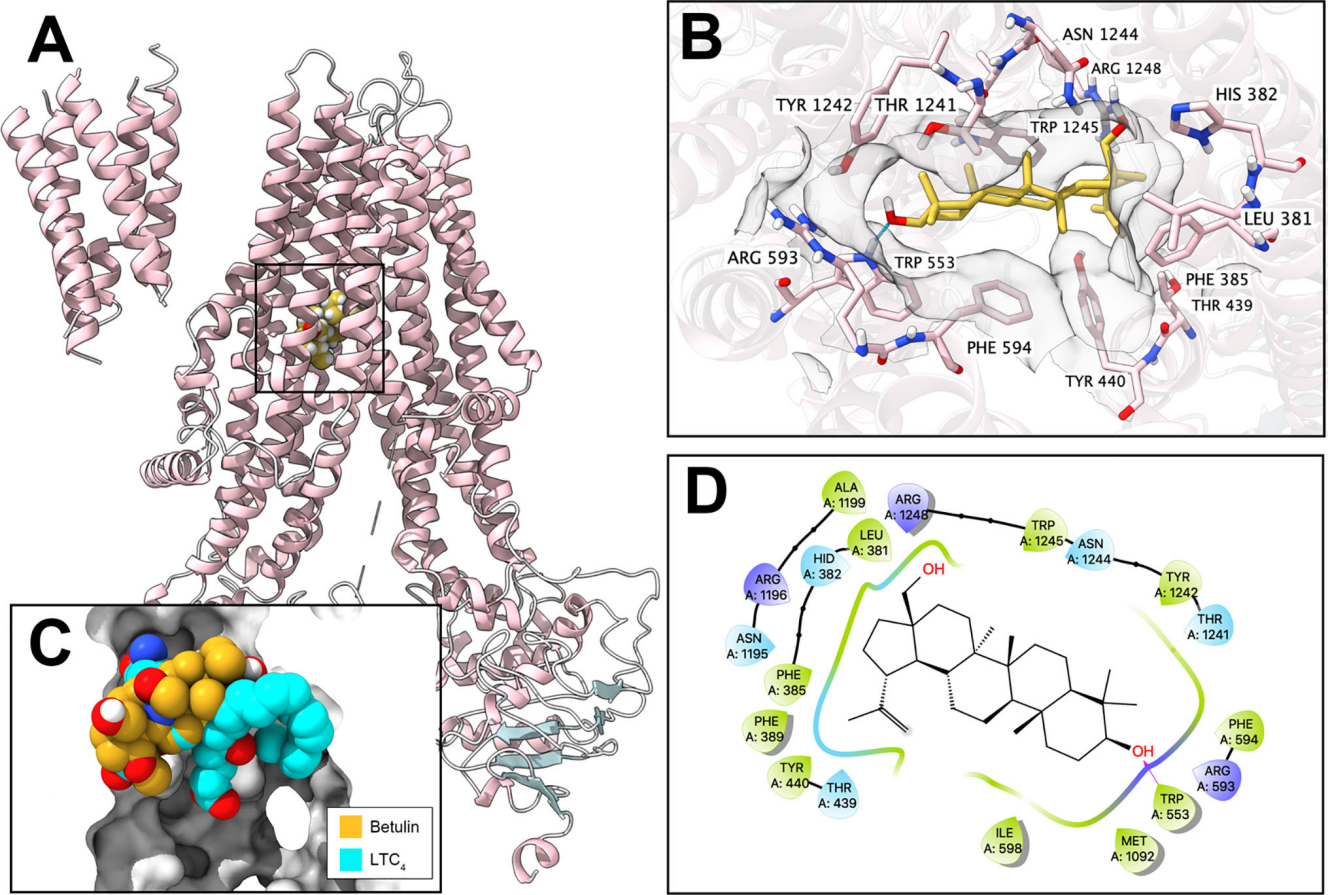
Interaction between betulin and human ABCC1 protein. **(A)** Overview of the best-scoring pose of betulin in the drug-binding pocket of ABCC1 protein (5UJA). ABCC1 was displayed as colored ribbons (helix: pink; strand: blue; coil: white). Betulin was displayed as colored balls. **(B)** Details of the interactions between betulin and ABCC1 (5UJA) binding pocket. ABCC1 helices were displayed as colored ribbons. Important residues were displayed as colored sticks (carbon: pink; oxygen: red; nitrogen: blue; hydrogen: white; fluoride: lime; chloride: light green). Surface formed by important residues were depicted as grey solid planes. Betulin was displayed as colored sticks (carbon: yellow; oxygen: red; nitrogen: blue; chloride: lime; fluoride: light green). Hydrogen bonds were displayed as blue dash lines. **(C)** Binding poses of betulin and ABCC1 substrate LTC_4_ in ABCC1 binding pocket. **(D)** 2D diagram of the interaction between betulin and ABCC1. Amino acids within 3 Å from betulin were displayed as colored bubbles (green: hydrophobic; blue: polar). Purple solid lines with arrow indicate hydrogen bonds.

To further validate the interaction of betulin and ABCC1 protein, an *in silico* analysis was conducted. Our results showed that betulin docked into the drug-binding site of ABCC1 with an affinity score of −6.8 kcal/mol. Overall, betulin binds in the pocket surrounded by the transmembrane domains of ABCC1 protein (**Figure 3A**), partially overlapping the leukotriene C_4_ (LTC_4_)-binding site (**Figure 3C**). Details of the ligand-receptor interaction are displayed in **Figure 3B**. The primary factor contributing to the binding affinity of betulin to the ABCC1 protein is *via* hydrophobic interactions. According to **Figures 3B, D**, betulin is positioned and stabilized in the hydrophobic cavity formed by Leu381, Phe385, Phe389, Tyr440, Thr439, Ile598, Phe594, Met1092, Thr1241, Tyr1242, Asn1244, and Tpr1245. Betulin was stabilized by a hydrogen bond formed with Trp553. Together, these results demonstrated that betulin has an interaction with the ABCC1 protein.

## Discussion

Nowadays, many natural-derived drugs serve as sources of novel drug discovery and are tested clinically (24, 25). Unfortunately, the efficacy of certain natural products could be compromised by MDR-associated ABC transporters (3, 4). ABCC1 (MRP1), ABCB1 (MDR1, P-glycoprotein/P-gp), and ABCG2 (BCRP/MXR) are extensively studies, and are commonly responsible for MDR (18, 19). Betulin and betulinic acid derived from birch bark have a broad spectrum of pharmacological activity (11, 26). Researchers reported that betulinic acid inhibits ABCB1-, ABCG2-, and ABCB5-mediated MDR with similar effectiveness as counterparts in parental cell lines (12). Also, Zhao et al. found that betulinic acid nanoparticle exerts its anticancer efficacy *via* downregulating ABCG1 oncogene expression level (13). These provide us a clue that betulin may interact with MDR-associated ABC transporters. However, the interaction of betulin and ABCC1 remains inconclusive and needs to be determined. Considering the similar cancer spectrum between ABCC1 and betulin as described in the introduction section, we herein focused on the effectiveness of betulin influenced by ABCC1 overexpression.

Our experiments started from an MTT-based cell viability assay to assess the cytotoxicity of betulin and a reference substrate drug vincristine with or without an inhibitor. The results indicated that ABCC1 overexpression can confer resistance to betulin in cancer cells expressing ABCC1. As KB-3-1 is a human epidermoid carcinoma cell line, it is possible that, to some extent, developing other mechanism induces drug resistance apart from ABCC1 overexpression in the KB-CV60 cell line. Hence, the cell viability of betulin was determined on ABCC1-transfected HEK293 cells. Similarly, betulin resistance was found in HEK293/ABCC1 cells as evidenced by the higher RF value in cells transfected with ABCC1 compared with its corresponding sensitive HEK293/pcDNA3.1 cells counterpart. Vincristine, a vinca alkaloid isolated from the Madagascar periwinkle *Catharanthus roseus* (27), served as a reference substrate of ABCC1 (2). Although betulin does not have as high of an RF value as vincristine, it is still rather comparable to vincristine. Importantly, betulin-conferred drug resistance can be antagonized by a known ABCC1 inhibitor, MK571. Overexpression of ABCB1 and ABCG2 is also central to MDR, therefore cell viability of cells overexpressing ABCB1 or ABCG2 was also examined after betulin treatment. Given different cellular context, it is possible that switching arginine to glycine (R > G) in the ABCG2 gene at amino acid 482 could affect substrate specificity and different resistance levels to substrate drugs (17). Our results showed that no significant difference in IC_50_ values was observed in drug-sensitive cells and corresponding MDR cells mediated by ABCB1, and wild-type or R482G-mutant ABCG2, which were consistent with results from Saeed et al (12).. Together, ABCC1 overexpression may promote betulin resistance, while overexpression of ABCB1 and ABCG2 could not confer resistance to betulin. Thus, we hypothesized that betulin is a, ABCC1 substrate.

Following mechanism-based studies, a Western blot analysis was conducted to assess the ABCC1 protein expression. Our results showed that within a 72 h incubation period, betulin upregulates ABCC1 expression level in time- and concentration-dependent manners. Interestingly, other researchers generated Pearson’s correlation coefficients (R-values) to correlate the expression level of different genes and the log_10_IC_50_ values for betulinic acid (12). Results from Saeed et al. demonstrated that there is no significant correlation between betulinic acid and ABCC1 gene expression. In the chemical structure, betulin and betulinic acid are different in hydroxymethyl and carboxyl groups, which may lead to differences in their pharmacological properties. We also postulated that this inconsistency may be the results from post-transcriptional and/or post-translational modification and regulation (28–31), which needs further validation.

Substrate drugs can occupy MDR-associated ABC transporters, and result in a competition with another drug substrate for transport function (20). As a result, a repurposed drug substrate acting as an inhibitor or a reversal agent has the ability to sensitize MDR-associated ABC transporter to another drug substrate (32). In our study, the [^3^H]-vincristine accumulation in cancer cells was measured. Results from an accumulation assay demonstrated that betulin at 25 μM inhibits MDR-mediated by ABCC1. However, the inhibitory effect of betulin to ABCC1 is not as strong as 25 μM MK571, which might be the result of high resistance level caused by ABCC1 in KB-CV60 cells and/or the lower RF value difference of betulin relative to vincristine between KB-3-1 cells and KB-CV60 cells. Notably, the concentrations of betulin used in the accumulation assay are higher than the IC_50_ values in corresponding cell lines, which can be toxic to the cells. However, the 4 h incubation of betulin with cells may not affect the cellular function. This is confirmed in the parental cells that no significant difference was observed between the vehicle group and the treatment groups. Of note, even the weaker inhibitory effect of betulin than MK571 counterpart, our findings do not warrant further investigation of betulin as a reversal agent or modulator to MDR mediated by ABCC1 overexpression.

The computational docking analysis serves as an efficient tool to predict the interaction of compound with protein models even though it indicates a virtual binding instead of an actual one (33, 34). Betulin received an affinity score of −6.8 kcal/mol with human ABCC1 protein model (5UJA). Also, it is known that leukotriene C_4_ (LTC_4_) can be pumped by ABCC1 (35). Our results revealed that betulin has binding interaction with human ABCC1 protein, and more importantly shares certain overlapping binding sites with LTC_4_. Overall, betulin may exhibit a similar substrate behavior.

## Conclusions

Betulin is susceptible to drug resistance mediated by ABCC1 overexpression, and a known ABCC1 inhibitor, MK571, can sensitize the cells expressing ABCC1 to betulin. ABCC1-induced resistance to betulin can be explained by its upregulated protein expression of ABCC1. Additionally, betulin at high concentration has the ability to inhibit ABCC1 transport function, which may affect the pharmacokinetic profile of other ABCC1 drug substrates, such as vincristine. These findings may be a valuable foundation for follow-up clinical investigation on the potential use of betulin.

## Data Availability Statement

The raw data supporting the conclusions of this article will be made available by the authors, without undue reservation.

## Author Contributions

Conceptualization: X-YC. Methodology: YY, X-YC, and J-QW. Writing-original draft: YY and X-YC. Writing-review and copyediting: X-YC, YY, Z-XW, and Z-SC. Supervision: JL and Z-SC. All authors contributed to the article and approved the submitted version.

## Funding

This research was funded by the National Natural Science Foundation of China (No.81973761).

## Conflict of Interest

The authors declare that the research was conducted in the absence of any commercial or financial relationships that could be construed as a potential conflict of interest.
